# Pre-pregnancy weight, the rate of gestational weight gain, and the risk of early gestational diabetes mellitus among women registered in a tertiary care hospital in India

**DOI:** 10.1186/s12884-023-05907-9

**Published:** 2023-08-15

**Authors:** Swapna Deshpande, Tarja I. Kinnunen, Anuradha Khadilkar, Jyothi Unni, Vandana Khanijo, Namrata Donga, Sangita Kulathinal

**Affiliations:** 1 Unit of Health Sciences, Faculty of Social Sciences, Tampere University, Tampere, Finland; 2 Paediatric Growth and Endocrine Department, Hirabai Cowasji Jehangir Medical Research Institute, Pune, India; 3 Department of Obstetrics and Gynaecology, Jehangir Hospital, Pune, India; 4 Department of Mathematics and Statistics, University of Helsinki, Helsinki, Finland

**Keywords:** Early GDM, Rate of gestational weight gain, Binomial regression, Interval censoring, India

## Abstract

**Background:**

The impact of pre-pregnancy weight and the rate of gestational weight gain (GWG) together on the risk of early GDM (< 24 weeks gestation; eGDM) has not been studied in the Indian context. We aimed to study the influence of (1) pre-pregnancy weight on the risk of eGDM diagnosed in two time intervals; and (2) in addition, the rate of GWG by 12 weeks on the risk of eGDM diagnosed in 19–24 weeks.

**Method:**

Our study utilized real-world clinical data on pregnant women routinely collected at an antenatal care clinic at a private tertiary hospital, in Pune, India. Women registering before 12 weeks of gestation (v_1_), with a singleton pregnancy, and having a follow-up visit between 19–24 weeks (v_2_) were included (*n* = 600). The oral glucose tolerance test was conducted universally as per Indian guidelines (DIPSI) at v_1_ and v_2_ for diagnosing eGDM. The data on the onset time of eGDM were interval censored; hence, we modeled the risk of eGDM using binomial regression to assess the influence of pre-pregnancy weight on the risk of eGDM in the two intervals. The rate of GWG by 12 weeks was added to assess its impact on the risk of eGDM diagnosed in v_2_.

**Result:**

Overall, 89 (14.8%) women (age 32 ± 4 years) were diagnosed with eGDM by 24 weeks, of which 59 (9.8%) were diagnosed before 12 weeks and 30 of 541 (5.5%) women were diagnosed between 19–24 weeks. Two-thirds (66%) of eGDM were diagnosed before 12 weeks of gestation. Women’s pre-pregnancy weight was positively associated with the risk of GDM in both time intervals though the lower confidence limit was below zero in v_1_. The rate of GWG by 12 weeks was not observed to be associated with the risk of eGDM diagnosed between 19–24 weeks of gestation. These associations were independent of age, height, and parity.

**Conclusion:**

Health workers may focus on pre-pregnancy weight, a modifiable risk factor for eGDM. A larger community-based study measuring weight and GDM status more frequently may be warranted to deepen the understanding of the role of GWG as a risk factor for GDM.

## Background

Gestational Diabetes Mellitus (GDM) is defined as any degree of glucose intolerance which is diagnosed for the first time during pregnancy, irrespective of treatment with diet or insulin [1]. GDM is associated with an increased risk of adverse maternal and fetal outcomes including preeclampsia, cesarean delivery, stillbirth, macrosomia, large for gestational age, and neonatal hypoglycemia [2–4]. Also, women with a history of GDM are at elevated risk of GDM in future pregnancies and the development of type 2 diabetes and cardiovascular disease in later life [5, 6]. The prevalence of GDM is increasing globally, also in India. There is a wide range of prevalence of GDM reported across India, varying from 3.8% in Kashmir to 35% in Punjab [7]. The increasing prevalence of GDM could be attributed to the increasing percentage of overweight and obesity that Indian women bear.

High maternal pre-pregnancy weight is a known risk factor for developing GDM [8–10]. Maternal height, a proxy to early life nutritional status and genetic makeup, is inversely associated with an increased risk of GDM [11–13]. To date, very few studies have demonstrated an association between the rate of gestational weight gain (GWG) prior to glucose screening and the incidence of GDM [14–16]. In principle, the rate of GWG should be considered until the onset of GDM only (not until screening) as later GWG does not affect the risk of developing GDM.

The Institute of Medicine (IOM), USA, published GWG guidelines that depend on pre-pregnancy body mass index (BMI) [17]. These recommendations are, however, meant for American women and therefore their applicability to Indian populations is unclear and limited by the fact that the BMI classification for Asians is different from the World Health Organization’s (WHO) general BMI classification. There are no national guidelines on GWG for Indian women.

As per the national guidelines for GDM diagnosis in India (Diabetes in pregnancy study group of India, DIPSI), women should be tested universally at the first antenatal visit and during the late second trimester, i.e., 24 to 28 weeks of gestation [18]. The early diagnosis of GDM is recommended because Indians are at a higher risk of developing diabetes. GDM diagnosed before 24 weeks of gestation fulfilling the criteria of standard GDM is termed ‘early GDM’ (eGDM) [19–21]. In the current study, we present the data on women’s eGDM status tested at two visits. These visits fell in two time-intervals during pregnancy: < 12 weeks, and between 19 and 24 weeks of gestation. It is to be noted that only the diagnosis times to eGDM are observed (< 12 weeks and between 19 to 24 weeks), and the onset times to eGDM are known to belong to either < 12 weeks or between 12 to 24 gestation weeks intervals only (interval censored). This is because the onset time to eGDM can be in an interval between the two visits when diagnosis is made. Thus, studying the impact of pre-pregnancy weight and rate of GWG on the risk of eGDM requires careful statistical consideration. Moreover, it has not been studied in the Indian context. Our aim was to study the influence of (1) pre-pregnancy weight on the risk of eGDM diagnosed in two-time intervals (before 12 weeks and between 19 and 24 weeks); and (2) in addition, the rate of GWG by 12 weeks on the risk of eGDM diagnosed in 19–24 weeks.

## Material and methods

### Study design and participants

Our study utilized routinely collected clinical data on pregnant women attending an antenatal care clinic at a tertiary-level hospital, in Pune, Maharashtra, India. Women who were registered at the hospital between January 2019 and December 2020, were within 12 weeks of gestation at the time of registration with a singleton pregnancy, had a visit during 19–24 weeks of gestation, and did not have any known comorbidities and medical conditions related to previous pregnancies were included in the study. Comorbidities included chronic respiratory illness, cardiovascular disease, gastrointestinal disease, hepatic disease, hypertension, diabetes mellitus, and medical conditions (including GDM) during any previous pregnancy, recurrent pregnancy loss, and medications that can affect weight gain. The trained staff in the hospital carefully examined previous medical reports, and blood sugar, HbA1C results if already done. All women underwent blood sugar testing according to DIPSI criteria at the first antenatal visit [18]. Women with blood sugar > 200 mg/dL were categorized as having diabetes already before pregnancy and they were asked to perform further investigations. These women were not included in the study. Women who did not have a visit between 19–24 weeks of gestation or had miscarriages or termination of pregnancy during early gestation were excluded from the analysis.

Ethics approval and consent to participate: The study was approved by the ethics committee of the hospital in Pune (Ethics Committee Jehangir Clinical Development Centre Pvt. Ltd., ECR/352/Inst /MH/2013/RR-16). All methods were performed in accordance with the relevant guidelines and regulations. Written informed consent was obtained from all participants.

### Sample size estimation

The sample size was estimated for exploring the association between pre-pregnancy BMI and the risk of GDM as previous studies on pre-pregnancy weight and risk of eGDM were not available. The sample size estimation was based on (1) the proportion of overweight or obese (23%) women of reproductive age in Pune [22], and (2) the risk of GDM among overweight/obese women (22.3%) and normal-weight women (8.5%) in a Pune-based study [23]. Sample size estimation and power calculation were performed using R (packages ‘EpiDisplay’ and ‘powerMediation’). Based on the sample size calculations, data on 423 women were required. We included data on about 600 women in the study to account for possible loss to follow-up.

### Measurements

Sociodemographic data and clinical history were collected at the first visit (before 12 weeks of gestation). These included age, education, parity, family history of diabetes (either/both parents), diabetes status, and self-reported pre-pregnancy weight. In addition, weight and height were measured by trained staff at the first visit. Height was measured with a stadiometer by measuring the maximum distance from the floor to the highest point on the head when the participant is facing directly ahead. Weight was measured in light clothing and without shoes using an electronic digital weighing machine. The BMI was calculated as the ratio of the weight in kilograms to the square of the height in meters (kg/m^2^). BMI at early pregnancy was classified according to WHO Asia Pacific BMI cut points [24]. Each participant’s weight was measured again at the second visit during 19 to 24 weeks.

The oral glucose tolerance test was conducted as per the DIPSI guidelines before 12 weeks of gestation and at a visit in the second trimester (between 19 to 24 weeks) for diagnosing eGDM status [18]. Fasting pregnant women were given 75 g oral glucose load, and if their 2-h plasma glucose was ≥ 140 mg/dL, they were diagnosed with eGDM. Participants with eGDM were treated by the hospital staff according to the usual standard of care practices.

### Statistical analysis

Continuous data are presented using mean (SD) or median (25^th^ and 75^th^ percentile), and categorical data are presented using frequencies (proportions). The first antenatal visit (before 12 weeks) is denoted as v_1_ and the second visit (between 19 to 24 weeks) as v_2_, throughout the manuscript. Total number of women with eGDM are presented by BMI categories for completeness, and to have a direct comparison with other reported studies. The GWG (kg) by v_1_ was calculated as the difference between the weight at v_1_ and the pre-pregnancy weight. GWG between the first and the second visits was calculated as the difference between the weight at v_1_ and v_2_. The rate of GWG (kg/week) at v_1_ was computed as the ratio of the GWG at v_1_ to gestational length in weeks at v_1_, and the rate of GWG (kg/week) at v_2_ as the ratio of GWG between v_1_ and v_2_ to the difference in gestational length at v_2_ and v_1_ in weeks. The proportions of women diagnosed with eGDM at v_1_ and v_2_ were also computed. We summarized the rate of GWG (kg/week) by v_1_ and v_2_ between three groups of women; (i) not diagnosed with eGDM; (ii) diagnosed with eGDM at v_1_, and (iii) diagnosed with eGDM at v_2_. We present distributions of maternal age, height, and weight at pre-pregnancy, at v_1_ and v_2_, and the rate of GWG at v_1_ and v_2_ using barcode plots. Each line in the barcode plots corresponds to a woman and hence, the plots are useful in visualizing the granularity of the data.

As described earlier, each woman was tested for eGDM status at v_1_ and v_2_. It is important to remember that only those who were tested negative for eGDM at v_1_ were tested at v_2_. No women were tested during 12–18 weeks of gestation for diagnosis of eGDM. As described in Background, the time of onset of eGDM was known to belong only to either before 12 weeks or 12–24 weeks’ time interval. Hence, the onset times were interval censored. A naive estimate of the total risk of eGDM by 24 weeks was calculated as the ratio of the total number of women diagnosed with eGDM to the total number of women tested for eGDM. Similarly, the risk of eGDM before 12 weeks was estimated as the ratio of the number of women diagnosed at v_1_ with eGDM to the number of women tested for eGDM at v_1_. The risk of eGDM in 12–24 weeks was estimated as the ratio of the number of women diagnosed with eGDM at v_2_ to the number of women tested for eGDM at v_2_. Thus, the risk of onset of eGDM in 12–24 weeks is the same as the risk of diagnosis of eGDM in 19 to 24 weeks, in the current setting.

We modeled the risk of eGDM using the diagnosis of eGDM before 12 weeks and between 19–24 weeks and binomial regression models to assess the influence of pre-pregnancy weight on the risk of eGDM [25]. Three models were implemented: Model 1 included an interval-specific intercept and no covariates. In Model 2, women’s age, parity, education, occupation, height, and family history of diabetes were added to Model 1. We added all covariates to Model 1 one by one, and then finalized the model based on the model’s AIC criterion (lowest AIC value). In Model 3, we included an additional covariate, the rate of GWG during early gestation (pre-pregnancy to v_1_), to the binomial models described above to assess the influence of pre-pregnancy weight and the rate of GWG by v_1_ on the risk of eGDM in 12–24 weeks (diagnosis between 19 to 24 weeks). We checked the linear relationship of the pre-pregnancy weight, and other continuous variables in relation to the risk of eGDM (logit scale) using restricted cubic spline transformation (‘rms’ package in R) [26]. The overall goodness of fit of the model was assessed visually using residual plots. The statistical analyses were performed in R (v4.0) and SAS v 9.4.

## Results

### Characteristics of the participants

Six hundred and fifty-four pregnant women consented to enroll in the study. Eight (1.2%) women were lost to follow-up due to relocation for delivery or discontinued the study. Of the 646 women, 7 (1.1%) had induced or spontaneous abortions during their early pregnancy. We present data on 600 women who enrolled in the study before 12 weeks of gestation and visited the clinic during 19 to 24 weeks. Women who had consented (*n* = 654) and those who are included in the analysis (*n* = 600) were not widely different in terms of their age, education, occupation, parity, and family history of diabetes.

Table 1 shows the baseline sociodemographic and anthropometric characteristics of 600 women with a mean age of 32 (SD 4) years. Of all women, 61% were nulliparous, 54% had completed post-graduate or professional education and 46% were homemakers.

**Table 1:** Baseline sociodemographic and anthropometric characteristics of women diagnosed with eGDM (before 12 weeks and in 19–24 weeks) and women without eGDM by 24 weeks

	eGDM diagnosed before 12 weeks *n* = 59	eGDM diagnosed in 19 to 24 weeks *n* = 30	No eGDM diagnosed by 24 weeks *n* = 511	Total *n* = 600	
	Mean (SD) or n (%)	Mean (SD) or n (%)	Mean (SD) or n (%)	Mean (SD) or n (%)	Median (q1, q3)
Age (years)	31.5 (4.0)	30.9 (3.3)	31.8 (4.1)	31.7 (4.0)	32.0 (29.0, 34.0)
Parity, n (%)					
0	33 (55.9%)	18 (60.0%)	317 (62.1%)	368 (61.4%)	
1	23 (39.0%)	12 (40%)	177 (34.6%)	212 (35.3%)	
≥ 2	3 (5.1%)	0	17 (3.3%)	20 (3.3%)	
Education, n (%)					
Up to 12th class	4 (6.8%)	1 (3.3%)	35 (6.9%)	40 (6.7%)	
Graduate / Diploma	27 (45.8%)	7 (23.4%)	202 (39.6%)	236 (39.2%)	
Post-Graduate or Professional	28 (47.6%)	22 (73.3%)	272 (53.1%)	322 (53.8%)	
Missing	0	0	2 (0.3%)	2 (0.3%)	
Occupation, n (%)					
Service	34 (57.6%)	13 (43.3%)	226 (44.1%)	273 (45.5%)	
Business / Self-employed	2 (3.4%)	6 (20.0%)	41 (8.0%)	49 (8.1%)	
Homemaker	23 (39.0%)	11 (36.7%)	242 (47.7%)	276 (46.0%)	
Missing	0	0	2 (0.3%)	2 (0.3%)	
Pre-pregnancy weight (kg)	61.6 (6.4)	64.6 (7.1)	60.0 (8.8)	60.4 (8.6)	59.0 (55.0, 65.0)
Weight measured at the first visit (kg)	62.4 (6.4)	66.0 (7.8)	60.8 (9.1)	61.2 (8.9)	60.0 (56.0, 66.0)
Height measured at the first visit (cm)	155.0 (4.8)	157.2 (6.2)	156.6 (4.9)	156.5 (5.0)	156.0 (153.0, 160.0)
BMI at the first visit (kg/m^2^)	25.9 (2.1)	26.7 (3.4)	24.8 (3.4)	25.0 (3.4)	24.5 (23.0, 26.5)

Mean (SD) and Median (q1, q3) or n (%)

The mean length of gestation was 8.0 weeks (SD 1.7) at v_1_ and 21.4 weeks (SD 1.2) at v_2_. Seven (1.2%) women were underweight, 142 (23.8%) were in the normal weight category, 339 (56.4%) were overweight and 112 (18.6%) were obese. Eighty-nine women (14.8%) were diagnosed with eGDM by 24 weeks. When comparing the risk of eGDM between the BMI categories, none of the underweight women, 4 (2.7%) of the normal weight women, 64 (18.9%) of the overweight women and 21 (18.8%) of the obese women were diagnosed with eGDM.

### Age, height, pre-pregnancy weight, and weight change by eGDM status

**Fig. 1:**
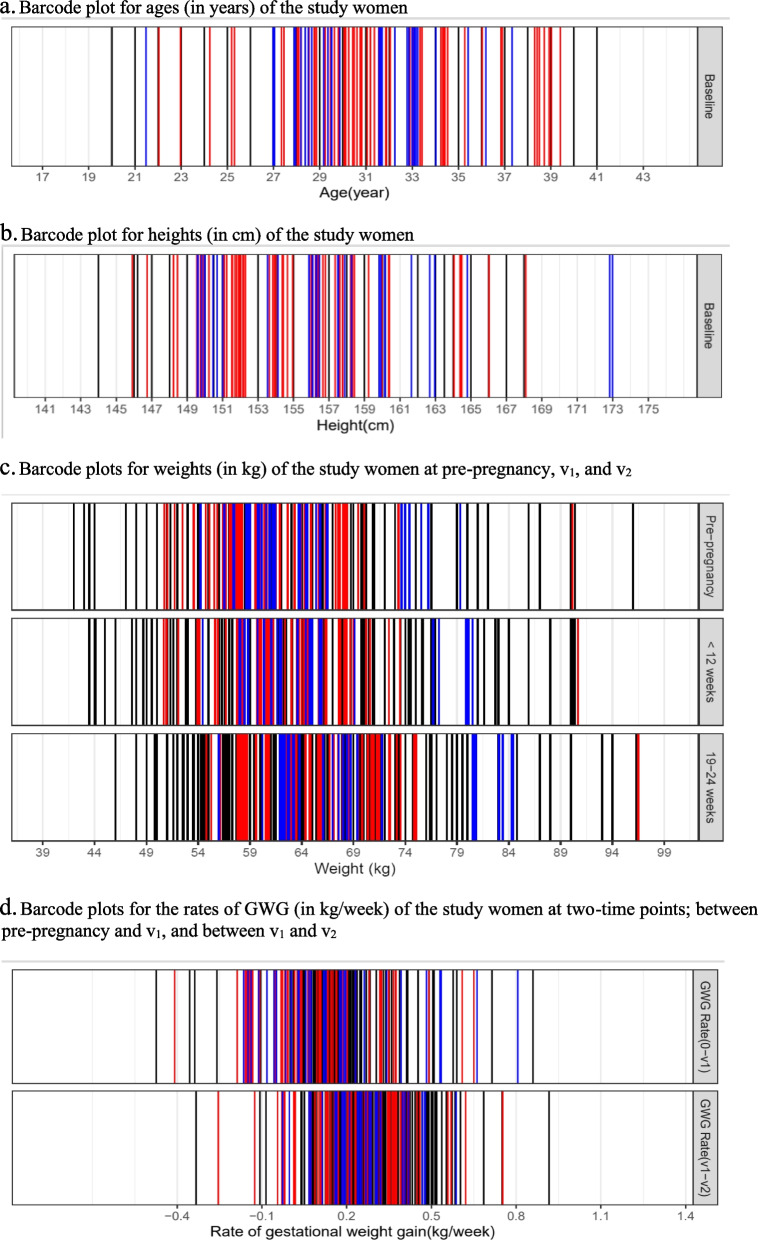
Barcode plots for age, height, weight at pre-pregnancy, and weight and rate of weight change at v_1_ (< 12 weeks) and v_2_ (19–24 weeks). Each bar represents a woman. Red bars correspond to women diagnosed with eGDM at v_1_, blue bars to women diagnosed with eGDM at v_2_, and black bars to women not diagnosed with eGDM

Figure 1a and b represent barcode plots for the maternal age and height at baseline. Each bar represents one woman. The age distribution is dense around 27 to 34 years and the distribution of height is dense around 150 to 158 cm. More red bars (indicating women diagnosed with eGDM at v_1_) are observed in the upper region of the age distribution and blue bars (indicating women diagnosed with eGDM at v_2_) towards the lower region of the distribution. On the other hand, blue bars are dense in the upper region of height distribution compared to the red bars. Thus, most women diagnosed with eGDM at v_1_ are likely to be older and shorter compared to women diagnosed with eGDM at v_2_.

Figure 1c represents weight at pre-pregnancy, v_1_, and v_2_. Figure 1d represents the rate of GWG from pre-pregnancy to v_1_ and from v_1_ to v_2_. The weight at pre-pregnancy is densely placed around 57 to 62 kg. The rate of GWG is distributed densely around 0 to 0.20 kg/week at v_1_ and 0.25 to 0.60 kg/week at v_2_. There are more women who were diagnosed with eGDM at v_2_ who were heavier at pre-pregnancy and had a higher rate of GWG at v_1_ than among women diagnosed with eGDM at v_1_.

### Pre-pregnancy weight and the rate of GWG among women with and without eGDM

Fifty-nine (9.8%) women were diagnosed with eGDM at v_1_. The mean pre-pregnancy weight was 61.6 (6.4) kg among women with eGDM and 60.3 (8.0) kg among women without eGDM. The mean rate of GWG by v_1_ was 0.074 (0.150) kg/week among women with eGDM at v_1_ and 0.070 (0.159) kg/week among women without eGDM. Out of 541 women who did not have eGDM at v_1_, 30 (5.5%) women were diagnosed with GDM at v_2_. Their mean pre-pregnancy weight was 64.6 (7.1) kg. The mean pre-pregnancy weight was 60.2 (8.6) kg in women without GDM at v_2_. The mean rate of GWG by v_1_ was 0.154 (0.169) kg/week among women with GDM at v_2_ and 0.066 (0.160) kg/week among women without GDM at v_2_. The mean rates of GWG from v_1_ to v_2_ were similar in both groups (0.249 and 0.256 kg/week, respectively).

### Pre-pregnancy weight, rate of GWG, and the risk of eGDM using binomial regression models

Table 2 represents estimates of and 95% confidence intervals for the regression parameters of binomial regression models. Based on the findings of the linearity tests, all regression models included linear association of continuous variables on the logit scale.

**Table 2:** Estimates of regression coefficients (95% CI) for the risk of eGDM in the binomial regression models

Covariates	Regression coefficient (95% CI)	*P* value
**Model 1**		
Time interval of v_1_ (< 12 weeks)	-2.218 (-2.496, -1.958)	< 0.001
Time interval of v_2_ (19–24 weeks)	-2.837 (-3.227, -2.487)	< 0.001
**Model 2**		
Time interval of v_1_ (< 12 weeks)	6.170 (-0.905, 13.297)	0.090
Time interval of v_2_ (19–24 weeks)	3.583 (-3.674, 10.849)	0.332
Pre-pregnancy weight * Time interval of v_1_	0.031(-0.010, 0.061)	0.055
Pre-pregnancy weight * Time interval of v_2_	0.063 (0.025, 0.098)	< 0.001
Age	-0.035 (-0.089, 0.019)	0.199
Parity		
Nulliparous	Ref	
Multiparous	0.187 (-0.270, 0.637)	0.417
Height	-0.059 (-0.107, -0.012)	0.014
**Model 3**		
Time interval of v_1_ (< 12 weeks)	5.86 (-1.226, 12.991)	0.110
Time interval of v_2_ (19–24 weeks)	3.42 (-3.907, 10.747)	0.359
Pre-pregnancy weight* Time interval of v_1_	0.030 (-0.002, 0.062)	0.059
Pre-pregnancy weight* Time interval of v_2_	0.057 (0.018, 0.093)	0.003
Age	-0.034 (-0.088, 0.021)	0.222
Parity		
Nulliparous	Ref	
Multiparous	0.192 (-0.266, 0.643)	0.408
Height	-0.057 (-0.105, -0.010)	0.018
Rate of weight change in early gestation (< 12 weeks)	1.700 (-0.231, 3.469)	0.072

Binomial regression Modeling

*CI* Confidence interval, *GDM* Gestational diabetes mellitus

**Fig. 2:**
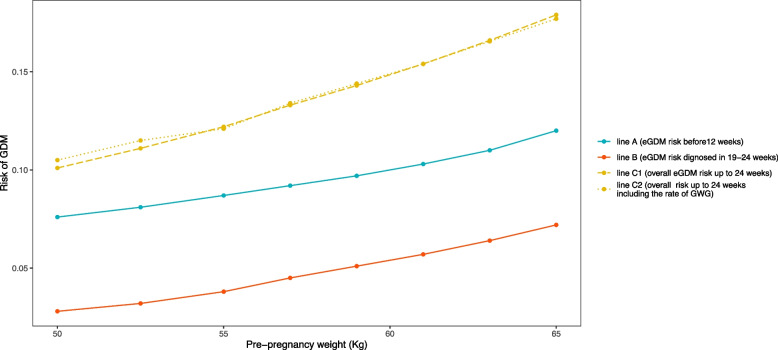
Estimated risk of eGDM under different scenarios of pre-pregnancy weight and rate of GWG using estimates in the regression models. Legend: Age = 30 years, height 155 cm, nulliparous, and rate of weight change of 0.10 kg/week by v_1_ (from pre-pregnancy to 12 weeks of gestation). Blue and orange lines show the estimated risk of eGDM by 12 weeks, and eGDM diagnosed in 19 to 24 weeks, respectively, under Model 2. The upper yellow long-dashed and dotted lines show the overall risk of eGDM by 24 weeks under models without and with the rate of gestational weight gain by 12 weeks under Models 2 and 3 respectively. The models were adjusted for maternal age, height, and parity

The risk of eGDM before 12 weeks was 9.8% and the risk of eGDM between 19 to 24 weeks was 5.5%. The overall risk of eGDM by the end of 24 weeks was 14.8%. Note that these are simple proportions of eGDM based on the diagnosis of eGDM in the two-time intervals before 24 weeks of gestation. In Model 2, women’s pre-pregnancy weight was positively associated with the risk of eGDM in both intervals. The risk of eGDM was lower in the interval 19 to 24 weeks compared to that before 12 weeks when adjusted for all covariates. For example, a 30-year-old nulliparous woman, with 60 kg of pre-pregnancy weight and 155 cm of height (BMI of 25 kg/m2) had 10% risk of getting diagnosed with eGDM before 12 weeks. The risk of eGDM diagnosis in 19 to 24 weeks for the same woman was 5.4% given she was not diagnosed with eGDM during the first visit leading to 15% overall risk of eGDM by the end of 24 weeks. For a woman with the same BMI of 25 kg/m2, but with the weight of 64 kg and height of 160 cm, the estimated risk of eGDM before 12 weeks reduced to 8.6%. Model 3 additionally included the rate of GWG by v1. The risk of eGDM diagnosed between 19 to 24 weeks and overall risk of eGDM by the end of 24 weeks did not increase in Model 3 compared with Model 2. This is further illustrated in Fig. 2 which represents the estimation of the risk of eGDM in the first interval (< 12 weeks) and in the second interval (19 to 24 weeks) by increasing pre-pregnant weight at a given age (30 years), parity (nulliparous) and height (155 cm). Line A (blue line) and B (orange line) present the risk of eGDM in the first interval and in the second interval, respectively, using Model 2. Lines C1 (yellow long-dashed) and C2 (yellow dotted) show the overall risks of eGDM by the end of the second interval without (Model 2) and with the rate of GWG by 12 weeks (Model 3). The Fig. 2 shows that the risk of GDM by the end of 24 weeks did not change due to the inclusion of the rate of GWG by 12 weeks. The models were adjusted for maternal age, height, and parity. The overall goodness of fit of each model was accepted.

## Discussion

In our study, nearly 15% of women were diagnosed with eGDM by the end of 24 weeks of gestation. Around 9.8% were diagnosed before 12 weeks and 5.5% were diagnosed during 19 to 24 weeks, thus the majority (59 out of 89, 66%) of eGDM were diagnosed before 12 weeks of gestation. The percentage of eGDM was similar among overweight and obese women. Women’s pre-pregnancy weight was positively associated with the risk of GDM in both time intervals though the lower confidence limit was just below zero in v_1_. The rate of GWG by 12 weeks was not associated with the risk of eGDM diagnosed between 19 to 24 weeks of gestation. These associations were also independent of maternal age, height, and parity. This finding implied that pre-pregnancy weight played a major role in the risk of development of eGDM.

We decided to keep pre-pregnancy weight and height as two separate explanatory variables to assess their individual and the combined role in the risk of eGDM. Maternal height was also associated with the risk of eGDM as already seen in other studies [10–12]. The risk of eGDM was slightly lower among taller women compared to shorter ones when pre-pregnancy weight, maternal age, and parity remained the same. For example, under Model 2, for a nulliparous woman of age 30 years and pre-pregnancy weight 60 kg, the risk of eGDM reduces from 19 to 11% when the height changes from 150 to 160 cm.

A wide range of prevalence (1%-23%) of eGDM has been reported from the different parts of the globe [27]. The percentage of eGDM diagnosed before 24 weeks in a large Irish cohort and Australian cohort were 19% and 17% respectively. Our study is one of the few studies from Asia reporting the incidence of eGDM. A study in Iran reported 10% of prevalence in early gestation (< 12 weeks) [28]. A review article by S Bhattacharya et. al. discussed the association between early GDM and standard GDM [20]. Many observational studies, except a few early studies, have demonstrated a positive association between eGDM and the standard GDM [20, 29]. Around one third of women diagnosed with GDM were diagnosed during the first trimester in a large Pan-India study [30]. A narrative review of observational studies reported that a high proportion (up to 70%) of women can be diagnosed with GDM in early pregnancy (either at the first antenatal visit or < 14 weeks) [31]. This has usually resulted in early initiation of treatment and preventing adverse pregnancy outcomes, though not always [31]. Several researchers also studied the impact of diagnosis and treatment of eGDM on maternal and neonatal outcomes. Recently published results of a randomized control trial have shown a modestly lower incidence of adverse neonatal outcomes among women treated for eGDM than those who received deferred or no treatment [21]. Our study showed a positive relationship between pre-pregnancy weight and the risk of eGDM. Since there are no guidelines developed for GWG for Indian or Asian women, we added the rate of GWG in early gestation (< 12 weeks) for exploring a possible relationship with the risk of eGDM diagnosed during 19 to 24 weeks. The rate of GWG by 12 weeks was not associated with the risk of eGDM that diagnosed between 19 to 24 weeks, which may be due to the small rate of GWG. Additionally, the pre-pregnancy weight was self-reported, which might have induced bias. Therefore, this result should be interpreted with caution. A recently published community-based study in China reported that early pregnancy BMI was associated with the risk of GDM, while BMI gain before GDM screening had no impact on the risk of GDM [32]. However, some studies in Asia and western countries have reported that women with a higher GWG in early gestation were at higher risk of developing GDM later compared to those with lower GWG [15, 16, 33]. A higher age is a known risk factor for GDM [34, 35]. As seen in the barcode plot (Fig. 1b), women who were diagnosed with eGDM by 12 weeks were more likely to be older than women diagnosed with eGDM during 19–24 weeks of gestation.

All women attending the antenatal clinic underwent screening for eGDM in our study, regardless of their BMI. The percentage of eGDM was similar in overweight and obese women (19% in both groups). A similar finding was observed in large population-based studies from Punjab, India, and in a national study using NFHS 2015–2016 data [36, 37]. In these studies, the risk of GDM was similar between overweight and obese women though higher than among the normal weight women.

### Strengths and weaknesses

One of the main strengths of this study is the universal screening for GDM of all women twice during early pregnancy. To our knowledge, this is the first study from India to present data on the incidence of eGDM. This study explored the relationship between pre-pregnancy weight and the rate of GWG in early gestation, and the risk of eGDM. The aspect of temporality is very important from the point of causality and seemed to be missing in many of the previous studies. We utilized routinely collected, real world clinical data to assess the risk of eGDM using sound statistical methodology. Due to the spread of visit weeks before 12 weeks and then between 19 to 24 weeks, we adapted the ‘interval censored’ analysis approach and employed binomial regression model to estimate the risk of eGDM. We see this as an appropriate analytical approach to studying such follow-up data where the diagnosis time is observed sparsely, and the onset time is known to fall in an interval between the consecutive visits. Our analysis which includes maternal height adds to the existing literature related to the association between maternal height and the risk of eGDM. Important covariates were presented using barcode plots which are better data visualization tools (presentation of data for each subject) than just presenting data using summary statistics. The universal screening at the first antenatal visit was performed per the DIPSI guidelines. The prevalence of GDM is expected to be higher when universal screening is applied than when screening risk groups alone, which should be kept in mind when comparing results between studies.

The major limitation of the study was the lack of oral glucose tolerance test data during 24 to 28 weeks of pregnancy as recommended by the DIPSI guidelines. Most of the women in this study visited the clinic earlier than 24 weeks (mean 21 weeks with SD 1.2 weeks and median 22 weeks). Therefore, we were not able to examine ‘Standard GDM’ or the risk of GDM for the entire pregnancy period. We used self-reported pre-pregnancy weight which has a chance of reporting bias. However, most of the women were educated (at least graduates), so it was likely that they were aware of their pre-pregnancy weight although they might still have misreported it. The rate of GWG in early gestation was not associated with the risk of GDM during 19 to 24 weeks in our study. This result should be interpreted with caution. Only two measurements of weight by 24 weeks may not be adequate to capture the dynamics of weight change during the pregnancy prior to the screening for eGDM. In this study, eGDM cases were diagnosed using DIPSI criteria, which are based on a fairly simple, non-fasting, single-test method. Even though IADPSG criteria are recommended, the DIPSI criteria are still being used in South Asian countries like India. The use of DIPSI criteria may miss some eGDM cases due to their low sensitivity [38, 39].

## Conclusion

An increase in the pre-pregnancy weight of a woman increases the risk of eGDM. Around two thirds of eGDM were diagnosed by 12 weeks of gestation. We did not observe an association between the rate of GWG in early gestation and the risk of eGDM diagnosed during 19 to 24 weeks of gestation. The pre-pregnancy weight played a major role in the risk of development of eGDM. There is a need for healthcare workers to focus on pre-pregnancy weight which is a key risk factor for GDM. A larger community-based study measuring weight and GDM status more frequently may be warranted to deepen the understanding of the role of GWG as a risk factor for GDM.

## Data Availability

The datasets used and analyzed during the current study are available from the corresponding author upon reasonable request. The data are not publicly available due to data privacy restrictions.

## Abbreviations

AIC: Akaike Information Criterion
BMI: Body mass index
DIPSI: Diabetes In Pregnancy Study group of India
GDM: Gestational diabetes mellitus
GWG: Gestational weight gain
IOM: Institute of Medicine
WHO: World health organization
